# Global Burden of Diseases Attributable to Early Maturation in 2021

**DOI:** 10.1002/mco2.70681

**Published:** 2026-03-15

**Authors:** Yujie Xu, Xueting Liu, Yidi Wang, Changxiao Xie, Siquan Zhou, Ye Tian, Jingyuan Xiong, Guo Cheng

**Affiliations:** ^1^ Laboratory of Molecular Translational Medicine Center for Translational Medicine Children's Medical Key Laboratory of Sichuan Province Key Laboratory of Birth Defects and Related Diseases of Women and Children (Sichuan University), Maternal & Child Nutrition Center, West China Second University Hospital, Sichuan University Chengdu China; ^2^ Healthy Food Evaluation Research Center West China School of Public Health West China Fourth Hospital, Sichuan University Chengdu China; ^3^ Healthy Promotion and Food Nutrition & Safety Key Laboratory of Sichuan University, West China School of Public Health West China Fourth Hospital Sichuan University Chengdu China

**Keywords:** age‐standardized incidence, early maturation, global burden, population attributable fraction

## Abstract

Early maturation poses a considerable health challenge worldwide. We aim to quantify the global disease burden attributable to early maturation in 2021. We calculated sex‐specific and region‐specific population attributable fractions (PAFs) of diseases through meta‐analysis and Mendelian randomization. The age‐standardized incidence rate (ASIR) with 95% uncertainty intervals (UIs) of these diseases, stratified by sex, age, and development status were estimated using the Global Burden of Diseases database. Asthma, Type 2 diabetes, ischemic heart disease, stroke, uterine cancer, testicular cancer, and depression was causally related with early maturation, with PAF ranging from 7.7% for uterine cancer to 0.8% for Type 2 diabetes. About 11.2 million new attributable cases occurs, with an ASIR of 210.1 (95% UI 155.1–280.7) cases per 100,000, with higher PAF in females (3.4%) than in males (2.3%). The ASIR was highest in North America (405.5, 309.0–526.3 cases per 100,000) and lowest in East Asia and the Pacific (120.8, 89.6–160.3 cases per 100,000). More developed regions showed 1.4 times higher incidence burden (ASIR 268.5, 199.6–356.6 cases per 100,000) than less developed regions. Therefore, regional adoption of effective public health interventions to alleviate early maturation, notably for females in more developed regions, has enormous potential to reduce worldwide disease burden.

## Graphical abstract

Early maturation significantly contributed to the global burden of seven major non‐communicable diseases, accounting for an estimated 11.2 million new cases worldwide in 2021. Our analysis revealed that this burden disproportionately affected females and populations in more developed regions. These specific findings provide a crucial evidence base for informing and tailoring future public health strategies and preventive interventions.

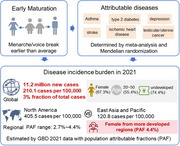

## 1 Introduction

Early puberty timing has profound implications for later life health [1], and the burden attributable to early maturation is expected to further increase in the coming decades due to the global trend of decreasing ages of puberty timing [2]. Globally, the age of puberty onset decreased by 0.24 years, almost 3 months per decade from 1977 to 2013 [2]. As such, prevention of early maturation could potentially moderate this increase and have substantial effect on disease prevention [3].

Understanding the burden attributable to early maturation is vital in prioritizing resources and developing effective preventative measures. A useful statistic to quantify this impact is the population attributable fraction (PAF), defined as the proportion of disease cases that would not occur in a population if a specific exposure was avoided. Data on the prevalence of early puberty timing and the magnitude of their associations with specific diseases are essential elements in estimating PAF. Although multiple meta‐analyses [4, 5, 6] and Mendelian randomization (MR) [7, 8, 9] have elucidated the relative risk (RR) of Type 2 diabetes [4, 9], cardiovascular disease [5, 7], and cancer [6, 8], further research is needed to update causal associations of early maturation with a broader spectrum of disorders.

Recent advances in data availability now enable a comprehensive assessment. The expanding collection of country‐level data on the prevalence of early maturation, combined with improved disease burden estimates from the Global Burden of Diseases (GBD), Injuries, and Risk Factors Study [10], has created an unprecedented opportunity to quantify its global impact. In addition, the estimation of regional‐specific PAF holds the potential to identify regional disparities in attributable burden due to various genetic predispositions, health behaviors, and healthcare practices, resulting in specific priority targets in particular regions.

To address these needs, our study aims to systematically estimate the incident cases attributable to early maturation among adults, providing essential evidence for targeted prevention strategies. Using the GBD database, epidemiological studies, and genome‐wide association study (GWAS) data, we expand early maturation–disease associations, along with their corresponding RRs, to calculate PAF for individual diseases at both a global and regional level and further estimated incident cases attributable to early maturation among adults aged 20 years and older in 2021.

## 2 Results

### 2.1 Causal Associations and PAFs

Our analysis identified seven diseases with significant causal links to early maturation (Table S1). The MR analysis showed that a 1‐year delay in pubertal age was associated with risk reductions ranging from 5.3% for major depression to 18.7% for Type 2 diabetes, with intermediate effect sizes observed for asthma (5.4%), ischemic heart disease (6.7%), stroke (11.2%), endometrial cancer (15.8%), and testicular cancer (16.1%).

**FIGURE 1:**
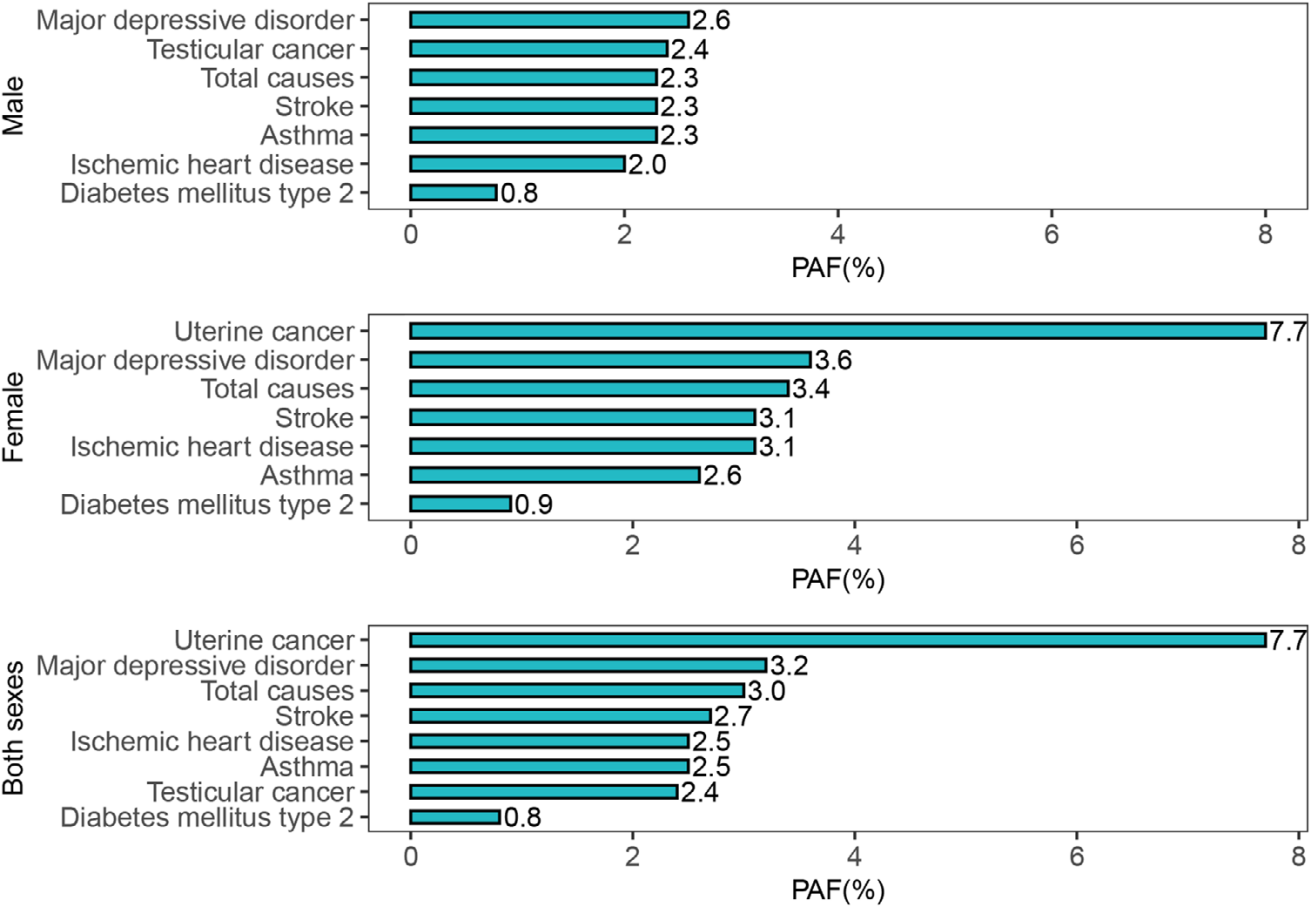
Estimated proportion and number of incident disease cases attributable to early maturation in adults aged 20 years and older worldwide in 2021, by sex. PAF, population attributable fraction.

Based on these disease‐specific RRs, the variation in PAF is shown by sex and disease type. As shown in Figure 1, the proportion of cases attributable to early maturation ranged from 2.6% for depression to 0.8% for Type 2 diabetes in males, and from 7.7% for uterine cancer to 0.8% for Type 2 diabetes in females. The overall PAF in females exceeded that in males by 1.5 times (3.4% vs. 2.3%).

### 2.2 Global Burden of Attributable Incidence

The overall and individual disease burden attributable to early maturation is presented in Table 1. In 2021, an estimated 11.2 million new cases in adults aged 20 years and older were attributable to early maturation with an ASIR of 210.1 (95% UI 155.1–280.7) cases per 100,000 person‐years. Depression was the leading contributor, accounting for 9.6 million cases (ASIR 181.3 cases per 100,000). This is followed by ischemic heart disease (792.8 thousand cases, ASIR 13.5), stroke (313.3 thousand cases, ASIR 5.3), and asthma (306.3 thousand cases, ASIR 5.7). The remaining 231.0 thousand cases were attributed to Type 2 diabetes, testicular cancer, and uterine cancer. Females experienced a higher incidence burden, constituting 7.5 million cases (67.3%, ASIR 278.5 cases per 100,000), compared to 3.7 million cases in females (32.8%, ASIR 140.1 cases per 100,000). Table 2 shows a detailed breakdown of these attributable cases by age group and development status. Around 6.2 million (55.4% of the 11.2 million total cases) cases attributable to early maturation occurred in people under 50 years old. However, we observed the largest number of attributable cases of ischemic heart disease, stroke, and uterine cancer in individuals aged 70 years and older. This distribution reflects the substantial contribution of depression, which predominantly affects younger age groups, alongside diseases like ischemic heart disease that peak later in life. Furthermore, 8.3 million (74.4%) cases occurred in less developed regions.

**TABLE 1:** Number of new cases in 2021 attributable to early maturation by sex and specific diseases, age ≥ 20 years.

	Total		Female		Male	
	Attributable cases, in thousands (95% UI)	ASIR (95% UI)	Attributable cases, in thousands (95% UI)	ASIR (95% UI)	Attributable cases, in thousands (95% UI)	ASIR (95% UI)
Asthma	306.3 (256.7, 359.0)	5.7 (3.4, 8.7)	182.1 (152.3, 213.8)	6.7 (3.9, 10.2)	124.2 (104.5, 145.1)	4.7 (2.8, 7.1)
Type 2 diabetes	192.3 (175.8, 209.7)	3.6 (2.7, 4.5)	101.9 (93.1, 111.1)	3.7 (2.9, 4.7)	90.4 (82.7, 98.7)	3.4 (2.6, 4.3)
Ischemic heart disease	792.8 (653.7, 954.7)	13.5 (8.9, 19.3)	432.9 (356.6, 521.7)	13.4 (8.8, 19.4)	359.9 (297.1, 433.0)	13.3 (8.9, 18.9)
Stroke	313.3 (280.2, 349.8)	5.3 (4.2, 6.7)	172.5 (154.8, 191.7)	5.3 (4.2, 6.8)	140.7 (125.5, 158.0)	5.2 (4.1, 6.6)
Testicular cancer	2.1 (1.9, 2.3)	0.04 (0.03, 0.05)	—	—	2.1 (1.9, 2.3)	0·08 (0.07, 0.09)
Uterine cancer	36.6 (32.6, 40.6)	0.6 (0.5, 0.7)	36.6 (32.6, 40.6)	1·2 (1.1, 1.4)	—	—
Depression	9566.3 (8143.5, 11,335.5)	181.3 (135.3, 240.7)	6613.3 (5634.6, 7843.8)	248.0 (184.7, 329.8)	2953.0 (2508.9, 3491.8)	113.4 (85.0, 150.2)
Total causes	11,209.6 (9544.4, 13,251.6)	210.1 (155.1, 280.7)	7539.2 (6423.9, 8922.7)	278.5 (205.5, 372.3)	3670.4 (3120.5, 4329.0)	140.1 (103.4, 187.2)

*Note*: The number of cases has been rounded to one decimal digit. Age‐standardized rate of incidence (ASIR) is expressed by cases per 100,000 person‐years.

Abbreviation: UI, uncertainty interval.

**TABLE 2:** Number of new cases in 2021 attributable to early maturation by development status and age groups, age ≥ 20 years.

	Number attributable to early maturation, by development status, in thousands (95% UI)		Number attributable to early maturation, by age group, in thousands (95% UI)		
	More developed regions^a^	Less developed regions	20–50 years	50–69 years	≥ 70 years
Asthma	105.4 (86.9, 124.9)	200.9 (169.9, 234.1)	162.4 (91.6, 253.3)	97.3 (60.7, 142.6)	46.6 (28.6, 67.2)
Type 2 diabetes	51.9 (48.0, 56.2)	140.3 (127.8, 153.6)	86.5 (63.9, 112.4)	92.6 (73.7, 113.0)	13.2 (9.7, 17.4)
Ischemic heart disease	181.9 (153.4, 216.3)	610.8 (500.2, 738.3)	91.6 (55.1, 137.6)	337.1 (218.2, 494.0)	364.0 (253.4, 504.4)
Stroke	73.5 (66.0, 81.9)	239.8 (214.3, 267.9)	44.0 (33.5, 57.2)	113.0 (86.4, 145.9)	156.2 (124.9, 192.7)
Testicular cancer	1.0 (0.9, 1.1)	1.1 (1.0, 1.2)	1.8 (1.6, 2.0)	0.3 (0.2, 0.3)	0.08 (0.07,0.1)
Uterine cancer	24.9 (23.0, 26.3)	11.7 (9.6, 14.3)	4.2 (3.6, 5.0)	21.6 (19.2, 24.4)	10.7 (9.1, 12.0)
Depression	2427.4 (2089.0, 2818.7)	7138.9 (6054.4, 8516.8)	5834.1 (4271.2, 7891.9)	2749.3 (2145.3, 3497.0)	982.9 (737.1, 1281.5)
Total causes	2866.0 (2467.3, 3325.3)	8343.6 (7077.1, 9926.3)	6224.7 (4520.4, 8459.3)	3411.2 (2603.7, 4417.1)	1573.8 (1162.9, 2075.3)

*Note*: The number of cases has been rounded to one decimal digit.

Abbreviation: UI, uncertainty interval.

^a^ Total for all countries in Europe and North America, Japan, Australia, and New Zealand.

### 2.3 Geographical Burden of Attributable Incidence

The geographical distribution of disease burden attributable to early maturation is presented in Table 3. North America (PAF 4.4%) and Sub‐Saharan Africa (3.7%) had the highest proportion of total cases attributable to early maturation, while South Asia (2.3%) and East Asia and the Pacific (2.7%) had the lowest. This trend was mirrored in the ASIR for overall attributable diseases, which was highest in North America (405.5 cases per 100,000), followed by Sub‐Saharan Africa (338.6 cases per 100,000), South Asia (198.3 cases per 100,000), and East Asia and the Pacific (120.8 cases per 100,000). In absolute terms, South Asia and East Asia and the Pacific each accounted for about 2.3 million cases. Notably, China and India harbored 1.5 and 1.7 million cases in 2021, with ASIRs of 118.8 and 191.6 cases per 100,000 person‐years, respectively. The United States had the highest national ASIR (417.9 cases per 100,000), with 985.6 thousand cases.

**TABLE 3:** Number of new cases in 2021 attributable to early maturation by geographical regions, age ≥ 20 years.

	Attributable cases in thousands (95% UI)	PAF (%)	ASIR per 100,000 (95% UI)
East Asia and Pacific	2319.9 (1993.4, 2714.9)	2.7	120.8 (89.6, 160.3)
China	1483.5 (1259.7, 1749.9)	2.8	118.8 (87.7, 157.6)
Europe and Central Asia	1701.8 (1465.3, 1975.6)	2.9	227.2 (166.6, 305.7)
Latin America and Caribbean	1215.8 (1045.0, 1445.0)	3.4	268.6 (200.6, 356.4)
Middle East and North Africa	947.8 (770.5, 1156.4)	3.2	324.5 (231.2, 449.7)
North America	1067.6 (924.3, 1231.2)	4.4	405.5 (309.0, 526.3)
USA	985.6 (854.2, 1136.2)	4.3	417.9 (318.7, 540.6)
South Asia	2274.2 (1934.7, 2706.5)	2.3	198.3 (147.7, 262.5)
India	1692 (1438.7, 2005.3)	2.3	191.6 (142.7, 252.9)
Sub‐Saharan Africa	1682.6 (1411.2, 2022.0)	3.7	338.6 (247.2, 456.1)
More developed regions^a^	2866.0 (2467.3, 3325.3)	3.4	268.5 (199.6, 356.6)
Less developed regions	8343.6 (7077.1, 9926.3)	2.8	200.4 (147.5, 268.5)
World	11,209.6 (9544.4, 13,251.6)	2.9	210.1 (155.1, 280.7)

*Note*: Regions were based on the World Bank regions, and the number of cases has been rounded to one decimal digit.

Abbreviations: ASIR, age‐standardized rate of incidence; PAF, population attributable fraction; UI, uncertainty interval.

^a^ Total for all countries in Europe and North America, Japan, Australia, and New Zealand.

### 2.4 Regional and Sex‐Specific Variations

**FIGURE 2:**
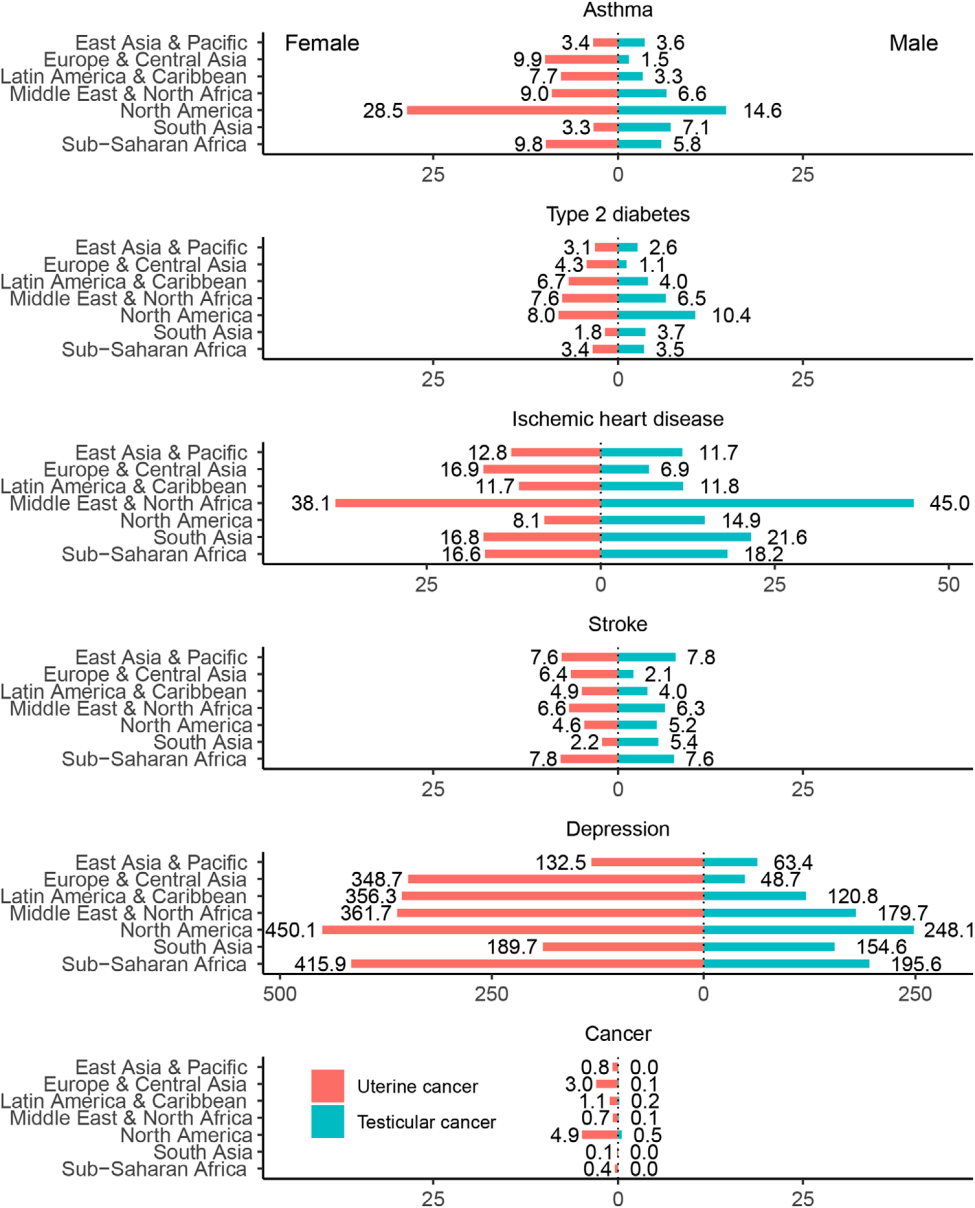
Age‐standardized rate (ASIR) of incidence per 100,000 person‐years of early maturation‐attributable diseases in 2021 in seven geographical regions by sex.

Figure 2 depicts individual disease burden attributable to early maturation stratified by sex and geographical regions. North America carries a substantial early maturation‐attributable disease burden, primarily driven by the highest ASIR of depression globally (450.1 cases per 100,000 in females and 248.1 cases per 100,000 in males), alongside the highest ASIR of asthma (28.5 cases per 100,000 in females and 14.6 cases per 100,000 in males). Other notable burdens included high incidence of ischemic heart disease in the Middle East and North Africa (38.1 cases per 100,000 in females and 45.0 cases per 100,000 in males) and high ASIR of stroke (7.6 cases per 100,000 in females and 7.8 cases per 100,000 in males) in East Asia and the Pacific. Most regions showed a higher ASIR in females for both individual and total attributable diseases, except South Asia, where a greater burden in males was observed (excluding depression).

**FIGURE 3:**
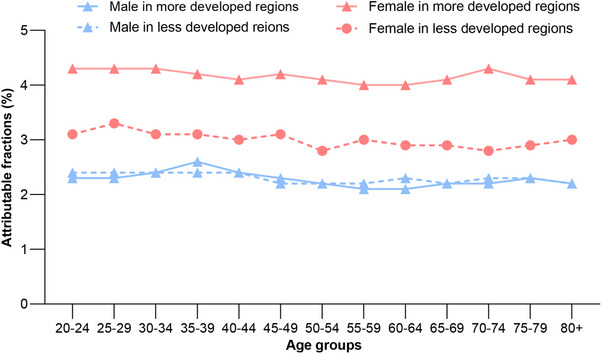
The proportion of new disease cases in 2021 attributable to early maturation, by sex, age group, and development status. More developed regions included all countries in Europe and North America, Japan, Australia, and New Zealand.

Finally, Figure 3 shows sex‐specific PAF patterns by development status. The PAF was slightly higher in more developed regions (3.4%) than in less developed regions (2.8%). A similar pattern was reflected in the ASIR (268.5 vs. 200.4 cases per 100,000). PAFs were consistently higher in females than in males across all regions. The lowest overall PAF was observed in males from less developed regions (about 2.0%), while females in more developed regions presented the highest PAF, exceeding the male groups by roughly double and surpassing females in less developed regions by 1.3 times.

## 3 Discussion

To our knowledge, this study is the first to estimate the global disease burden attributable to early maturation. Estimated 11.2 million global cases were attributable to early maturation in 2021 with 67.3% occurring in females. PAFs ranged from 7.7% for uterine cancer to 0.8% for Type 2 diabetes. Females in more developed regions recorded the highest PAF followed by females in less developed regions.

This study used integrated methods across epidemiological and GWAS data, which expands the current evidence regarding the impact of early puberty timing [8, 11], and facilitated precise measurement of its attributable burden. There are various biological pathways underlying these associations. In terms of the impact of early maturation on cardiometabolic health, alterations in protein and lipid by sex hormones [12], and changes in DNA methylation might be involved [13]. Evidence shows that early exposure to gonadotropin or gonadotropin‐mediated signals could potentially trigger the development of germ cell tumors, such as testicular cancer or endometrial cancer. Brain maturation and psychosocial adjustment are influenced by the timing of puberty, resulting in the development of internalizing psychological symptoms, including depressive symptoms [14].

With a global ASIR of 210.1 cases per 100,000 person‐years attributable cases of diseases in 2021, it is evident that early maturation significantly contributes to the global disease burden. The PAF of early maturation for diseases ranged from 0.8% to 7.7% across specific diseases (with an average of 3% overall), which is comparable to other reported modifiable factors. For instance, 5.6% of cancer cases were attributable to alcohol consumption [15], and attributable cases of outdoor air pollution contributed to 1.8%–6.4% of total cases [16]. Notably, the window period between early maturation in adolescence and the development of diseases might last years to decades, and the estimated disease burden in our current study resulted from early maturation exposure specifically during the years from the 1960s to 2000s. Considering the accelerating pace of early puberty timing over the last two decades, 2000–2020 [2], this attributable fraction and disease burden are projected to rapidly increase in the near future. From a public health standpoint, it is thus urgent to adopt measures against this phenomenon.

Of these early maturation‐attributable diseases, depression bears the greatest burden with the highest ASIR and the largest number of absolute cases. Depression is a leading cause of disability around the world, affecting people across the life span [17]. It is estimated that 5% of adults suffer from this disorder, but more than 75% of them do not receive any treatment [18]. Childhood incidence also increases sharply around the age of 13 years in girls and 16 years in boys and continues to rise into young adulthood [19]. With the World Health Organization's Comprehensive Mental Health Action Plan 2013–2030 [20], preventing early maturation might provide a new approach to alleviate the global burden of depression. Other attributable diseases, especially ischemic heart disease and stroke [21, 22], incur substantial economic burdens to society and increase mortality or disability, thus even a minor decline in the prevalence of early maturation could have profound public health implications in reducing their overall health burden.

Disease burden attributable to early maturation varied substantially across geographical regions. The highest ASIR is presented in North America, followed by Sub‐Saharan Africa. Since the late 19th century, trends of early maturation have been initially reported in high‐income countries in North America, contributing to the largest incidence burden of this region cumulatively. The highest PAF in North America (4.4%) and the higher disease burden in the United States also demonstrated this hypothesis. Contrary to North America, the high burden in Sub‐Saharan Africa is complicated. Since countries in this region are currently experiencing rapid urbanization, lifestyle transition, and inadequate health care expenditure, a high burden might be a consequence of other exposures, for example, the pandemic of obesity and chronic disease [23, 24]. In addition, due to limited data on early maturation prevalence in this region, we estimated the prevalence using risk‐based pooling methods. More cautions should be made regarding our findings.

We found that more developed world regions experienced a greater burden attributable to early maturation than less developed regions, along with higher PAF observed in such regions. Similar to the North American situation, changes in diet consumption (e.g., increased affordability of energy‐dense foods), food systems (e.g., more industrialized supply systems), and lifestyle factors (e.g., reduced physical activity and sedentary lifestyles) have first occurred in developed countries during the 1960s–2000s [25, 26], leading to much earlier increase in the prevalence of early puberty timing in these regions than less developed regions. Potential intervention strategies could include promoting childhood physical activity and nutritional education, both of which have shown promise in moderating pubertal timing, particularly in high‐burden settings where these modifiable factors may have the greatest impact. Larger absolute attributable numbers and lower ASIR in less developed regions, such as China in East Asia and the Pacific and India in South Asia, are likely indicative of the impact of population size. Reducing the incidence of early maturation in these areas could potentially be a more efficient approach to decreasing disease cases. With rapid development and lifestyle transition, less developed countries have recently experienced a faster decline in puberty timing than developed regions in the past [27]. This shift in trend implies that previous approaches, which have been proven effective in more developed regions, could not be directly extrapolated to less developed regions.

It is worth noting that the disease burden attributable to early maturation is primarily found in females, predominantly those in more developed regions. Historical records dating back to the 19th century indicate a higher prevalence of early puberty timing and even precocious puberty among females than males [28]. Possible reasons for sex differences are not yet fully comprehended; however, recent findings imply that it could be linked to genetic vulnerability, adiposity, and hormone sensitivity [29].

This study has some important strengths. We have first quantified the global burden attributable to early maturation from an overall perspective and have identified health inequalities across regions. To be specific, we underscore greater attention to the attributable burden in North America and more developed regions, and to females at the population level.

Several limitations should be addressed. First, even though the representativeness of the available data is theoretically acceptable, the use of aggregated country‐level data does not fully represent the entire region. Due to the inconsistent availability of large population‐based surveys on early maturation across countries, we included as much published data as possible from around the world. We prioritized population‐based studies with large sample sizes and high representativeness, and applied geographical pooling methods to synthesize an average value that best reflects regional prevalence. Given that geographic regions often reflect shared ancestry, population genetics, and adaptations to regional environments shaped by natural selection, as well as similar climatic conditions, ecosystems, and dietary habits [30, 31, 32], pubertal development among children within the same region tends to exhibit relative homogeneity. Nevertheless, regional differences and the uneven distribution of other factors influencing pubertal development cannot be overlooked. Further research is needed to develop a clearer understanding of early maturation, particularly in countries that lack large‐scale child cohort studies.

Second, due to limited data, some commonly used assumptions in disease burden estimates might introduce uncertainty [33, 34]. For instance, we assume that the link between early puberty and disease outcomes is consistent across regions. This was necessary because region‐specific risk estimates are scarce, making geographic comparisons difficult. To ensure consistency, we used a meta‐analytic approach to calculate average risk estimates and applied a uniform RR globally as a practical solution. Future research should focus on generating region‐specific data to improve accuracy. Meanwhile, since early puberty occurs in childhood, but related health issues often appear decades later, typically in adulthood or mid‐life, we assumed a 20‐year lag between exposure and outcome. This allowed us to estimate the 2021 disease burden caused by early maturation in the past. However, this assumption may not fully reflect individual differences or life course influences, which could affect the precision of these estimates.

Third, although puberty timing may have changed over time, we assumed it stayed mostly stable from the 1960s to the 2000s to allow for broader data collection across countries. Research shows a slow decline in the age of puberty during this time, about 0.4 years over 40 years, especially in boys, compared to a faster decline of 0.5 years per decade after 2000 [2]. This assumption was pragmatically necessary to combine different historical datasets but may have caused some timing errors. Future studies should use age‐period‐cohort models to better understand these changes over time.

Finally, despite disparities among regional or sex‐specific attributable burdens being helpful for policymakers, we could not explore the underlying socioeconomic mechanisms limited by the data and study scope. Furthermore, due to the limitations of our meta‐analytic approach, we were unable to assess potential non‐linear associations between early puberty and disease risk. Future research should explore possible non‐linear associations, which could offer a more accurate and nuanced understanding of how early pubertal timing impacts long‐term health outcomes.

## 4 Conclusions

Early maturation contributes substantially to the global disease burden, which was greater among females in more developed regions. Our regional‐specific and sex‐specific findings can serve as a useful reference for informing policies and programs more effectively.

## 5 Materials and Methods

### 5.1 Study Design

This study used mixed methods to take advantage of worldwide evidence and data. Through literature review and meta‐analyses of published findings, we identified the prevalence of early maturation at the national level and their associations with several disorders. Our attributable burden estimation was only based on disorders causally associated with early maturation, as determined by MR analysis of GWAS data. We further calculated attributable fractions for each disease and applied these fractions to worldwide disease incidence data from GBD 2021 (available at https://vizhub.healthdata.org/gbd‐results/). This study complies with GATHER recommendations [35], which can be found at the GATHER website. All analyses were conducted using Stata (Version 15.0), except for the MR analysis, which was conducted with R (Version 4.2). Associations with a *p* value of less than 0.05 were regarded as statistically significant.

### 5.2 Definition of Early Maturation

Early maturation refers to the condition where a child experiences physical and hormonal changes related to pubertal growth at a younger age than average [36]. These changes include breast development and menarche in girls and testicular growth and voice break in boys. Since puberty timing varies across genetic background and environmental exposure, there is no universally accepted age cutoff standard for defining early maturation. In epidemiological studies, early maturation is commonly defined as the occurrence of menarche or voice break before reaching the age at which 95% of the reference population has experienced these pubertal milestones. During 1960s–2000s, western counties especially North America and Europe, reported a median age of 12 years at menarche, while Asian countries generally reported later median ages ranging from 13 to 14 years. According to this published evidence, we defined early maturation as menarche age before 12 years [37] (before 13 years for Asians) [38] for girls and age at voice break earlier than the population average for boys [11].

### 5.3 Geographical Areas

Global estimates of incident cases attributable to early maturation were determined for the seven geographical regions, which were categorized by the World Bank regions and used by the GBD database. These regions include East Asia and Pacific, Europe and Central Asia, Latin America and Caribbean, Middle East and North Africa, North America, South Asia, and Sub‐Saharan Africa. We also calculated separate incidence estimates for China, India, and the United States due to their large population sizes within their respective regions. According to the UN classification system based on basic economic status by the World Economic Situation and Prospects (WESP) [39], countries were further grouped into (1) more developed regions (i.e., developed economies in WESP): countries in Europe, North America, as well as Australia, New Zealand, and Japan, and (2) less developed regions (encompassing economies in transition, and developing economies in WESP): all other countries.

### 5.4 Source of Early Maturation Prevalence and Relative‐Risk Data

Sources of data used for PAF calculation are summarized in Table S2. As early maturation occurs in childhood and its clinical outcomes manifest decades later in midlife, a considerable lag period is required between exposure and outcome assessment in order to reduce temporal bias. According to the widely used time‐lagged exposure prevalence approach in this field, we assumed a minimum 20 years of lagged exposure prevalence to estimate the disease burden attributable to early maturation. We thus obtained sex‐specific, country‐specific prevalence of early puberty timing between the 1960s and 2000s from population‐based cohort surveys. The aforementioned cohorts have been published following rigorous peer review, thus ensuring the highest standards of quality. If multiple cohorts reported early maturation prevalence in the same country, we first pooled these data using the inverse variance meta‐analysis approach. Regional estimates for early maturation prevalence were calculated using geographical pooling methods when country‐level prevalence data were available in this region or risk‐based pooling methods if there was insufficient country‐level evidence [40]. We applied the geographical pooling approach by calculating a weighted average of prevalence estimates. The weights were generated by multiplying the sample size of these studies with the number of disease cases in each country as provided by GBD 2021. Only for early maturation prevalence in Sub‐Saharan Africa, we considered a risk‐based pooling method where a linear regression model was fitted with prevalence in known regions as the outcome and individual disease incidence as the predictor.

RR estimates were pooled by meta‐analysis of epidemiology studies, including cohort and case‐control designs. We estimated the odds ratio (OR), RR, or hazard ratio (HR) estimates with their 95% confidential intervals for each related disease, pooling using a fixed‐effect meta‐analysis if *I* ^2^ < 50 or a random‐effect meta‐analysis if *I* ^2^ ≥ 50% [41]. Through this manner, associations between early maturation and its related disease were validated across different ethnic groups. We further assumed that these links are constant worldwide and used a single pooling RR for each disease in the calculation of respective PAFs [42]. The exact search strategies and meta‐analysis details are provided in Table S3.

### 5.5 Causal Association Determination

We collected GWAS data from multiple datasets to assess causal links between early puberty timing and diseases (Table S4). Given the strong correlation in the genetic architecture for pubertal timing across sexes [43], we used single‐nucleotide polymorphisms (SNPs) as instruments that were associated with menarche age combining 329,245 women [44]. For male‐specific diseases, we employed significant SNPs of voice break, including 205,354 men as instruments [43]. Multiple approaches were implemented to undertake a bidirectional two‐sample MR analysis. We assessed OR for each disease associated with a 1‐year increase in puberty timing; thus, an OR < 1 indicates that earlier puberty timing acts as a risk factor of the disease.

### 5.6 Source of Disease Incidence Data

Estimated number of new cases attributable to early maturation in individuals aged 20 years and older in 2021 worldwide was obtained from the GBD 2021 report, stratified by regions, sex, and age groups (from 20 to 80 in 5‐year intervals, and 85 years and older). Uncertainty was derived for all estimates by simulating 1000 draws from each estimate's posterior distribution to calculate uncertainty arising from primary inputs, sample sizes in the data collected, adjustments made to the data during modeling, and model estimation [45]. The GBD database provided incidence estimates for 369 diseases in 204 countries and territories across seven regions. Since endometrial cancer, the most common subtype of uterine cancer, accounting for over 90% of all uterine cancer cases [46], is unavailable in the GBD dataset, we analyzed the disease burden of uterine cancer as a suitable alternative. We calculated age‐standardized rates (ASIR) of incidence attributable to early maturation per 100,000 person‐year for each disease using the World Health Organization's standard population. The total number of attributable diseases in males and females was computed by summing disease cases over all age groups and individual diseases to obtain global sex‐specific cases and total cases.

### 5.7 Attributable‐Risk Calculation

The number of new cases attributable to early maturation was calculated by multiplying the worldwide incidence data by PAF. PAF is an estimate that relies on strong causal associations and combines the magnitude of the impact of exposure with its distribution in the population. We calculated age‐specific and region‐specific PAFs for each disease using the following Lavin‐based formula [47]:

$$\mathrm{PAF}=\frac{P_{e}\times \left(RR_{X}-1\right)}{P_{e}\times \left(RR_{X}-1\right)+1}$$

where *P_e_* is the prevalence of exposure in the general population and RR*_x_* is the RR associated with this exposure. These estimated numbers of cases have been reported in thousands and rounded to one decimal digit to avoid false precision.

## Author Contributions

G.C. and J.Y.X. conceived and designed this study. X.T.L. and Y.D.W. contributed to literature review and meta‐analysis. S.Q.Z. contributed to MR analysis. Y.J.X., C.X.X., and Y.T. contributed to data collection and data analysis. Y.J.X. wrote the original manuscript, and G.C. and J.Y.X. revised this manuscript. All authors have read and approved the final manuscript.

## Funding

This study was funded by the National Natural Science Foundation of China (grant nos. 82304135 and U25A20154) and the Department of Science and Technology of Sichuan Province (grant no. 2024NSFSC1806). The funder of the study had no role in study design, data collection, data analysis, data interpretation, or writing of the report.

## Ethics Statement

The authors have nothing to report.

## Conflicts of Interest

The authors declare no conflicts of interest.

## Supporting information




**Supporting File 1**: mco270681‐sup‐0001‐Tables.docx

## Data Availability

GBD data are available at https://vizhub.healthdata.org/gbd‐results/. Additional data are available through approval by the corresponding author.

## References

[1] J. Wise , “Puberty Timing Has Profound Effect on Later Health, Study Finds,” BMJ 350 (2015): h3318. 26092870 10.1136/bmj.h3318

[2] C. Eckert‐Lind , A. S. Busch , J. H. Petersen , et al., “Worldwide Secular Trends in Age at Pubertal Onset Assessed by Breast Development Among Girls: A Systematic Review and Meta‐Analysis,” JAMA Pediatrics 174, no. 4 (2020): e195881. 32040143 10.1001/jamapediatrics.2019.5881 PMC7042934

[3] F. F. Chen , Y. F. Wang , and J. Mi , “Timing and Secular Trend of Pubertal Development in Beijing Girls,” World Journal of Pediatrics 10, no. 1 (2014): 74–79. 24464668 10.1007/s12519-014-0456-2

[4] T. Cheng , F. R. Day , R. Lakshman , and K. K. Ong , “Association of Puberty Timing With Type 2 Diabetes: A Systematic Review and Meta‐Analysis,” PLoS Medicine 17, no. 1 (2020): e1003017. 31905226 10.1371/journal.pmed.1003017 PMC6944335

[5] K. Okoth , J. S. Chandan , T. Marshall , et al., “Association Between the Reproductive Health of Young Women and Cardiovascular Disease in Later Life: Umbrella Review,” BMJ 371 (2020): m3502. 33028606 10.1136/bmj.m3502 PMC7537472

[6] B. J. Fuhrman , S. C. Moore , C. Byrne , et al., “Association of the Age at Menarche With Site‐Specific Cancer Risks in Pooled Data From Nine Cohorts,” Cancer Research 81, no. 8 (2021): 2246–2255. 33820799 10.1158/0008-5472.CAN-19-3093 PMC8137527

[7] M. Cao and B. Cui , “Negative Effects of Age at Menarche on Risk of Cardiometabolic Diseases in Adulthood: A Mendelian Randomization Study,” Journal of Clinical Endocrinology & Metabolism 105, no. 2 (2020): 515–522. 10.1210/clinem/dgz071 31614369

[8] F. R. Day , D. J. Thompson , H. Helgason , et al., “Genomic Analyses Identify Hundreds of Variants Associated With Age at Menarche and Support a Role for Puberty Timing in Cancer Risk,” Nature Genetics 49, no. 6 (2017): 834–841. 28436984 10.1038/ng.3841 PMC5841952

[9] W. Xing , Q. Lv , Y. Li , et al., “Genetic Prediction of Age at Menarche, Age at Natural Menopause and Type 2 Diabetes: A Mendelian Randomization Study,” Nutrition, Metabolism and Cardiovascular Diseases 33, no. 4 (2023): 873–882. 10.1016/j.numecd.2023.01.011 36775707

[10] IHME , “Global Burden of Disease (GDB),” University of Washington, 2020, https://www.healthdata.org/research‐analysis/gbd.

[11] C. Minelli , D. A. van der Plaat , B. Leynaert , et al., “Age at Puberty and Risk of Asthma: A Mendelian Randomisation Study,” PLoS Medicine 15, no. 8 (2018): e1002634. 30086135 10.1371/journal.pmed.1002634 PMC6080744

[12] E. L. Ding , Y. Song , J. E. Manson , et al., “Sex Hormone‐Binding Globulin and Risk of Type 2 Diabetes in Women and Men,” New England Journal of Medicine 361, no. 12 (2009): 1152–1163. 19657112 10.1056/NEJMoa0804381 PMC2774225

[13] J. A. Resztak , J. Choe , S. Nirmalan , et al., “Analysis of Transcriptional Changes in the Immune System Associated With Pubertal Development in a Longitudinal Cohort of Children With Asthma,” Nature Communications 14, no. 1 (2023): 230. 10.1038/s41467-022-35742-z PMC9842661 36646693

[14] S. E. Mouridsen and F. W. Larsen , “Psychological Aspects of Precocious Puberty. An Overview,” Acta Paedopsychiatrica 55, no. 1 (1992): 45–49. 1310370

[15] F. Islami , A. Goding Sauer , K. D. Miller , et al., “Proportion and Number of Cancer Cases and Deaths Attributable to Potentially Modifiable Risk Factors in the United States,” CA: A Cancer Journal for Clinicians 68, no. 1 (2018): 31–54. 29160902 10.3322/caac.21440

[16] F. Valent , D. Little , R. Bertollini , L. E. Nemer , F. Barbone , and G. Tamburlini , “Burden of Disease Attributable to Selected Environmental Factors and Injury Among Children and Adolescents in Europe,” Lancet 363, no. 9426 (2004): 2032–2039. 15207953 10.1016/S0140-6736(04)16452-0

[17] C. D. Mathers and D. Loncar , “Projections of Global Mortality and Burden of Disease From 2002 to 2030,” PLoS Medicine 3, no. 11 (2006): e442. 17132052 10.1371/journal.pmed.0030442 PMC1664601

[18] COVID 19 Mental Disorders Collaborators , “Global Prevalence and Burden of Depressive and Anxiety Disorders in 204 Countries and Territories in 2020 Due to the COVID‐19 Pandemic,” Lancet 398, no. 10312 (2021): 1700–1712. 34634250 10.1016/S0140-6736(21)02143-7 PMC8500697

[19] A. S. F. Kwong , D. Manley , N. J. Timpson , et al., “Identifying Critical Points of Trajectories of Depressive Symptoms From Childhood to Young Adulthood,” Journal of Youth and Adolescence 48, no. 4 (2019): 815–827. 30671716 10.1007/s10964-018-0976-5 PMC6441403

[20] World Health Organization , “Comprehensive Mental Health Action Plan 2013–2030,” published 21 September, 2021, https://www.who.int/publications/i/item/9789240031029.

[21] A. E. Moran , M. H. Forouzanfar , G. A. Roth , et al., “Temporal Trends in Ischemic Heart Disease Mortality in 21 World Regions, 1980 to 2010: The Global Burden of Disease 2010 Study,” Circulation 129, no. 14 (2014): 1483–1492. 24573352 10.1161/CIRCULATIONAHA.113.004042 PMC4181359

[22] GBD 2019 Stroke Collaborators , “Global, Regional, and National Burden of Stroke and Its Risk Factors, 1990‐2019: A Systematic Analysis for the Global Burden of Disease Study 2019,” Lancet Neurology 20, no. 10 (2021): 795–820. 34487721 10.1016/S1474-4422(21)00252-0 PMC8443449

[23] NCD Risk Factor Collaboration (NCD‐RisC) , “Worldwide Trends in Body‐Mass Index, Underweight, Overweight, and Obesity From 1975 to 2016: A Pooled Analysis of 2416 Population‐Based Measurement Studies in 128·9 Million Children, Adolescents, and Adults,” Lancet 390, no. 10113 (2017): 2627–2642. 29029897 10.1016/S0140-6736(17)32129-3 PMC5735219

[24] H. N. Gouda , F. Charlson , K. Sorsdahl , et al., “Burden of Non‐Communicable Diseases in Sub‐Saharan Africa, 1990‐2017: Results From the Global Burden of Disease Study 2017,” Lancet Global Health 7, no. 10 (2019): e1375–e1387. 31537368 10.1016/S2214-109X(19)30374-2

[25] D. Talukdar , S. Seenivasan , A. J. Cameron , and G. Sacks , “The Association Between National Income and Adult Obesity Prevalence: Empirical Insights Into Temporal Patterns and Moderators of the Association Using 40 Years of Data Across 147 Countries,” PLoS One 15, no. 5 (2020): e0232236. 32401794 10.1371/journal.pone.0232236 PMC7219711

[26] B. A. Swinburn , G. Sacks , K. D. Hall , et al., “The Global Obesity Pandemic: Shaped by Global Drivers and Local Environments,” Lancet 378, no. 9793 (2011): 804–814. 21872749 10.1016/S0140-6736(11)60813-1

[27] T. Leone and L. J. Brown , “Timing and Determinants of Age at Menarche in Low‐Income and Middle‐Income Countries,” BMJ Global Health 5, no. 12 (2020): e003689. 10.1136/bmjgh-2020-003689 PMC7733094 33298469

[28] L. Xie , Q. Tang , D. Yao , et al., “Effect of Decaffeinated Green Tea Polyphenols on Body Fat and Precocious Puberty in Obese Girls: A Randomized Controlled Trial,” Frontiers in Endocrinology 12 (2021): 736724. 34712203 10.3389/fendo.2021.736724 PMC8546255

[29] S. J. Semaan and A. S. Kauffman , “Developmental Sex Differences in the Peri‐Pubertal Pattern of Hypothalamic Reproductive Gene Expression, Including Kiss1 and Tac2, May Contribute to Sex Differences in Puberty Onset,” Molecular and Cellular Endocrinology 551 (2022): 111654. 35469849 10.1016/j.mce.2022.111654 PMC9889105

[30] C. M. Beall , “Two Routes to Functional Adaptation: Tibetan and Andean High‐Altitude Natives,” Proceedings of the National Academy of Sciences of the United States of America 104, no. S1 (2007): 8655–8660. 17494744 10.1073/pnas.0701985104 PMC1876443

[31] S. J. Ulijaszek , “Human Eating Behaviour in an Evolutionary Ecological Context,” Proceedings of the Nutrition Society 61, no. 4 (2002): 517–526. 12691181 10.1079/pns2002180

[32] G. H. Perry , N. J. Dominy , K. G. Claw , et al., “Diet and the Evolution of Human Amylase Gene Copy Number Variation,” Nature Genetics 39, no. 10 (2007): 1256–1260. 17828263 10.1038/ng2123 PMC2377015

[33] GBD 2021 Adolescent BMI Collaborators , “Global, Regional, and National Prevalence of Child and Adolescent Overweight and Obesity, 1990‐2021, With Forecasts to 2050: A Forecasting Study for the Global Burden of Disease Study 2021,” Lancet 405, no. 10481 (2025): 785–812. 40049185 10.1016/S0140-6736(25)00397-6 PMC11920006

[34] GBD 2019 Dementia Forecasting Collaborators , “Estimation of the Global Prevalence of Dementia in 2019 and Forecasted Prevalence in 2050: An Analysis for the Global Burden of Disease Study 2019,” Lancet Public Health 7, no. 2 (2022): e105–e125. 34998485 10.1016/S2468-2667(21)00249-8 PMC8810394

[35] G. A. Stevens , L. Alkema , R. E. Black , et al., “Guidelines for Accurate and Transparent Health Estimates Reporting: The GATHER Statement,” Lancet 388, no. 10062 (2016): e19–e23. 27371184 10.1016/S0140-6736(16)30388-9

[36] S. A. Berenbaum , A. M. Beltz , and R. Corley , “The Importance of Puberty for Adolescent Development: Conceptualization and Measurement,” Advances in Child Development and Behavior 48 (2015): 53–92. 25735941 10.1016/bs.acdb.2014.11.002

[37] F. R. Day , C. E. Elks , A. Murray , K. K. Ong , and J. R. Perry , “Puberty Timing Associated With Diabetes, Cardiovascular Disease and Also Diverse Health Outcomes in Men and Women: The UK Biobank Study,” Scientific Reports 5 (2015): 11208. 26084728 10.1038/srep11208 PMC4471670

[38] E. Hwang , K. W. Lee , Y. Cho , H. K. Chung , and M. J. Shin , “Association Between Age at Menarche and Diabetes in Korean Post‐Menopausal Women: Results From the Korea National Health and Nutrition Examination Survey (2007‐2009),” Endocrine Journal 62, no. 10 (2015): 897–905. 26194132 10.1507/endocrj.EJ15-0192

[39] J. Bremner , A. Frost , C. Haub , M. Mather , K. Ringheim , and E. Zuehlke , “World Population Highlights: Key Findings From PRB's 2010 World Population Data Sheet,” Population Bulletin 65, no. 2 (2010): 1–12.

[40] C. de Martel , J. Ferlay , S. Franceschi , et al., “Global Burden of Cancers Attributable to Infections in 2008: A Review and Synthetic Analysis,” Lancet Oncology 13, no. 6 (2012): 607–615. 22575588 10.1016/S1470-2045(12)70137-7

[41] J. P. Higgins and S. G. Thompson , “Quantifying Heterogeneity in a Meta‐Analysis,” Statistics in Medicine 21, no. 11 (2002): 1539–1558. 12111919 10.1002/sim.1186

[42] W. Chen , C. Xia , R. Zheng , et al., “Disparities by Province, Age, and Sex in Site‐Specific Cancer Burden Attributable to 23 Potentially Modifiable Risk Factors in China: A Comparative Risk Assessment,” Lancet Global Health 7, no. 2 (2019): e257–e269. 30683243 10.1016/S2214-109X(18)30488-1

[43] B. Hollis , F. R. Day , A. S. Busch , et al., “Genomic Analysis of Male Puberty Timing Highlights Shared Genetic Basis With Hair Colour and Lifespan,” Nature Communications 11, no. 1 (2020): 1536. 10.1038/s41467-020-14451-5 PMC7093467 32210231

[44] A. Harroud , J. A. Morris , V. Forgetta , et al., “Effect of Age at Puberty on Risk of Multiple Sclerosis: A Mendelian Randomization Study,” Neurology 92, no. 16 (2019): e1803–e1810. 30894442 10.1212/WNL.0000000000007325 PMC6550505

[45] S. Feng , T. Wang , Y. Su , et al., “Global Burden, Risk Factors, and Projections of Early‐Onset Dementia: Insights From the Global Burden of Disease Study 2021,” Ageing Research Reviews 104 (2025): 102644. 39701185 10.1016/j.arr.2024.102644

[46] N. Galant , P. Krawczyk , M. Monist , et al., “Molecular Classification of Endometrial Cancer and Its Impact on Therapy Selection,” International Journal of Molecular Sciences 25, no. 11 (2024): 5893. 38892080 10.3390/ijms25115893 PMC11172295

[47] M. L. Levin , “The Occurrence of Lung Cancer in Man,” Acta ‐ Unio Internationalis Contra Cancrum 9, no. 3 (1953): 531–541. 13124110

