# Supplementary material for: Global Burden of Diseases Attributable to Early Maturation in 2021
Source: MedComm (2020). 2026 Mar 15;7(4):e70681. doi: 10.1002/mco2.70681 (PMC13042796; doi:10.1002/mco2.70681)
Supplement: Supplementary file 1 — Supporting File 1: mco270681‐sup‐0001‐Tables.docx [file MCO2-7-e70681-s001.docx]

**Global burden of diseases attributable to early maturation in 2021**

Yujie Xu^1^, Xueting Liu^2^, Yidi Wang^2^, Changxiao Xie^2^, Siquan Zhou^2^, Ye Tian^2^, Jingyuan Xiong^2,3^*, Guo Cheng^1^*

**Author Affiliations:**

^1^ Laboratory of Molecular Translational Medicine, Center for Translational Medicine, Children’s Medical Key Laboratory of Sichuan Province, Key Laboratory of Birth Defects and Related Diseases of Women and Children (Sichuan University), Maternal & Child Nutrition Center, West China Second University Hospital, Sichuan University, Chengdu, 610041, China;

^2^ Healthy Food Evaluation Research Center, West China School of Public Health and West China Fourth Hospital, Sichuan University, Chengdu, 610041, China;

^3^ Healthy Promotion and Food Nutrition & Safety Key Laboratory of Sichuan University, West China School of Public Health and West China Fourth Hospital, Sichuan University, Chengdu, 610041, China;

**Corresponding Author:**

Guo Cheng, PhD, 17 Renminnan Road 3^rd^ Section, West China Second University Hospital, Sichuan University, Chengdu 610041, China. E-mail: gcheng@scu.edu.cn; telephone: +86-28-8550-3675;

Jingyuan Xiong, PhD, 16 Renminnan Road 3^rd^ Section, West China School of Public Health and West China Fourth Hospital, Sichuan University, Chengdu 610041, China. Email: jzx0004@tigermail.auburn.edu.

**Supplemental Tables**

Table S1. Associations of early puberty timing and health outcomes.

Table S2. Details of early maturation prevalence data used in the calculation of population attributable fraction.

Table S3. Search terms to find relevant articles for inclusion in the meta-analysis.

Table S4. Information on GWAS datasets.

**Table S1. Associations of early puberty timing and health outcomes.**

| **Diseases** | **Meta-analysis^1^ of epidemiology data for relative risk** | | |  | **MR analysis^2^ of GWAS data for causal effects** | | |
| --- | --- | --- | --- | --- | --- | --- | --- |
|  | **No. study** | **OR/RR** | ***P*** |  | **No. SNPs** | **OR/RR** | ***P*** |
| Type 2 diabetes | 32 | 1.06 (1.01, 1.10) | 0.041 |  | 165 | 0.81 (0.75, 0.88) | <0.001 |
| Asthma | 13 | 1.17 (1.07, 1.28) | 0.025 |  | 174 | 0.95 (0.90, 0.99) | 0.019 |
| Breast cancer | 21 | 1.15 (1.04, 1.23) | 0.014 |  | 168 | 1.01 (0.96, 1.06) | 0.714 |
| Stroke | 14 | 1.20 (1.12, 1.29) | 0.038 |  | 173 | 0.89 (0.79, 0.99) | 0.047 |
| Prostate cancer | 6 | 1.21 (1.02, 1.43) | 0.002 |  | 74 | 0.94 (0.85, 1.05) | 0.251 |
| Testicular cancer | 8 | 1.22 (1.00, 1.50) | 0.044 |  | 75 | 0.84 (0.71, 0.99) | 0.042 |
| Ischemic heart disease | 8 | 1.19 (1.06, 1.33) | 0.036 |  | 172 | 0.93 (0.88, 0.99) | 0.024 |
| Depression | 9 | 1.22 (1.09, 1.35) | <0.001 |  | 170 | 0.95 (0.92, 0.97) | <0.001 |
| Endometrial cancer | 11 | 1.45 (1.23, 1.70) | <0.001 |  | 174 | 0.84 (0.78, 0.92) | <0.001 |

^1^Data were pooled using a random effect model of meta-analysis;

^2^Data were calculated by the primary method of MR analysis, except for testicular cancer and prostate cancer, the exposure of MR analysis was age at menarche; MR, GWAS, OR, odds ratio, RR, relative risk.

**Table S2. Details of early maturation prevalence data used in the calculation of population attributable fraction.**

| **Country** | **Region** | **n** | **Cohort** | **Prevalence in population** | **Time phase** |
| --- | --- | --- | --- | --- | --- |
| USA | North America | 100547 | Nurses’HealthStudy II | 24.30% | 1965-1970 |
| Germany | Europe | 1503 | KORA | 7.80% | 1985-1988 |
| China | East Asia | 121431 | REACTION | 18.90% | 1960s |
| China | East Asia | 7349 | Guangzhou Biobank Cohort Study | 6.30% | 1960-1965 |
| Korea | East Asia | 3254 | KNHANES IV (2007–2009) | 3.10% | 1960-1965 |
| Korea | East Asia | 12336 | Korean National Health and Nutrition Examination Survey | 5.20% | 1975-1980 |
| UK | Europe | 13308 | EPIC-Norfolk cohort study | 21.40% | 1960-1965 |
| Bangladesh | South Asia | 1535 | cohort | 31.80% | 1975-1980 |
| China | East Asia | 3304 | cohort | 12.60% | 1960-1970 |
| Brazil | Latin America | 8075 | ELSA-Brazil) | 7.20% | 1965-1973 |
| Korea | East Asia | 4326 | KNHANES IV | 5.70% | 1980-1985 |
| Sweden | Europe | 30697 | (BEST Gothenburg | 25.00% | 1960-1976 |
| Ten countries | Europe | 126721 | Inter LACE | 14.90% | 1965-1975 |
| Iran | North Africa and middle east | 5626 | TLGS | 16.20% | 1990-2000 |
| Japan | East Asia | 37511 | Japan Public Health Center-based Study | 26.50% | 1960s |
| UK | Europe | 250037 | The UK Biobank study | 4.30% | 1975-1980 |
| UK | Europe | 197714 | The UK Biobank study | 20.10% | 1975-1980 |
| China | East Asia | 16114 | Chinese cohort | 10.80% | 1978-1980 |
| Mexico | Latin America | 29335 | Mexican National Health Survey (2000) | 14.40% | 1960s-1980s |
| UK | Europe | 1217840 | The Million Women Study | 20.20% | 1961-1970 |
| Japan | East Asia | 37965 | The Japan Collaborative Cohort Study | 14.70% | 1960s |
| Singapore | East Asia | 34022 | The Singapore Chinese Health Study | 13.40% | 1960s |
| Finland | Europe | 3952 | NFBC 1966 | 12.20% | 1981-1983 |
| UK | Europe | 3648 | UK biobank | 11.10% | 1990-2000 |
| Korea | East Asia | 60119 | Health Examinees Study (HEXA) | 27.80% | 1975-1980 |
| Korea | East Asia | 945729 | National Health Insurance Sharing Service (NHISS) | 1.10% | 1965-1970 |
| USA | North America | 3711 | the Growing Up Today Study | 15.90% | 1996-2000 |
| USA | North America | 15674 | National Health and Nutrition Examination Survey (NHANES) | 21.70% | 1970-1980 |
| Canada | North America | 1176 | National Longitudinal Survey of Children and Youth | 16.80% | 1990-2000 |
| China | East Asia | 2291 | the Taiwan Children Health Study | 9.80% | 2000-2010 |
| China | East Asia | 2291 | the Taiwan Children Health Study | 11.60% | 2000-2010 |
| Sweden | Europe | 3461 | The Omega study (1996–2008) | 14.60% | 1975-1985 |
| UK | Europe | 1968 | 1958 British Birth Cohort | 16.00% | 1970s |
| Swiss | Europe | 2492 | SAPALDIA | 12.00% | 1980s |
| Brazil | Latin America | 1350 | The 1993 Pelotas (Brazil) birth cohort study | 15.00% | 2000s |
| UK | Europe | 239701 | UK biobank | 22.6% | 1975-1980 |
| Denmark | Europe | 10059 | the nationwide Danish Twin Registry | 9.30% | 1985-1990 |
| 10 countries | Europe | 3354 | the European Community Respiratory Health Survey II | 16.20% | 1960-1985 |
| UK | Europe | 243316 | UK biobank | 20.10% | 1975-1980 |
| UK | Europe | 192067 | UK biobank | 4.30% | 1975-1980 |
| USA | North America | 905 | CHS | 24.30% | 1990-2000 |
| Germany | Europe | 992 | ISAAC Phase II | 19.10% | 1990-1995 |
| Spain | Europe | 1335 | case-control | 18.10% | 1960-1970 |
| Indonesia | South Asia | 15744 | the Indonesian Family Life Survey | 5.80% | 1960-2000 |
| Korea | East Asia | 1224547 | the National Health Insurance Service data‑ base of Korea | 1.60% | 1960s |
| Korea | East Asia | 66104 | the Korean Heart Study | 21.20% | 1980s |
| USA\Australia\Canada\New Zealand | North America | 1128 | cohort | 40.30% | 1960-2000 |
| Denmark, France, Germany, Greece, Italy, the Netherlands, Norway, Spain, Sweden and United Kingdom | Europe | 302618 | the European Prospective Investigation into Cancer and Nutrition | 16.9% | 1960s |
| Iran | North Africa and middle east | 795 | cohort | 19% | 1970-1978 |
| Sri Lanka | South Asia | 415 | cohort | 13.30% | 1970s |
| Korea | East Asia | 66466 | Korean Heart Study (KHS) | 5.40% | 1965-1970 |
| USA | North America | 114409 | NIH-AARP Diet and Health Study | 47.30% | 1960s |
| Mexico | Latin America | 1146 | cohort | 12.90% | 1960s |
| Spain | Europe | 2433 | cohort | 26.30% | 1960s |
| Australia | East Asia | 2056 | cohort | 9.60% | 1960s |
| Finland | Europe | 11795 | FinRSPC | 17.40% | 1960s |
| USA | North America | 91961 | multiracial cohort | 21.80% | 1960s |
| USA | North America | 50884 | the Sister Study | 22.50% | 1965-1970 |
| USA | North America | 27536 | the Vitamins And Lifestyle cohort | 18.90% | 1960s |
| Japan | East Asia | 33410 | Three-Prefecture Cohort Study | 22.10% | 1960s |
| Mexico | Latin America | 113450 | The Mexican Teachers’ Cohort | 24.60% | 1975-1990 |
| Denmark | Europe | 23178 | The Danish Nurse Cohort | 19.20% | 1960s |
| China | East Asia | 11296 | cohort | 12.70% | 1960s |

**Table S3. Search terms to find relevant articles for inclusion in the meta-analysis.**

| **Database** | **Search terms** | **Retrieval** |
| --- | --- | --- |
| Type 2 diabetes mellitus | ((puberty[Title/Abstract]) OR (menarche[Title/Abstract])) OR (sexual Development[Title/Abstract]) OR (pubertal[Title/Abstract])) OR (spermarche[Title/Abstract]) OR (tanner[Text Word]) OR (voice break*[Text Word]) OR (sexual maturation[Text Word])) AND ((diabetes[Title/Abstract]) OR (diabetic[Title/Abstract]) OR (insulin[Title/Abstract]) OR (blood sugar[Title/Abstract]) OR (glucose[Title/Abstract]) OR (glycated[Title/Abstract]) OR (glycosylated[Title/Abstract])) AND ((“cohort Studies”[Mesh]) OR (cohort [Title/Abstract]) OR (prospective [Title/Abstract]) OR (longitudinal [Title/Abstract]) OR (“Epidemiologic studies”[Mesh]) OR (“case-control studies”[Mesh]) OR OR (Retrospective[Text Word]) OR (Recall*[Text Word]) OR (“Cross-sectional studies”[Mesh]) | 6236 |
| Stroke | ((puberty[Title/Abstract]) OR (menarche[Title/Abstract])) OR (sexual Development[Title/Abstract]) OR (pubertal[Title/Abstract])) OR (spermarche[Title/Abstract]) OR (tanner[Text Word]) OR (voice break*[Text Word]) OR (sexual maturation[Text Word])) AND (“Cardiovascular Disease”[Title/Abstract] OR “Heart disease”[Title/Abstract] OR “Peripheral arterial disease”[Title/Abstract] OR “Stroke”[Title/Abstract] OR “Cerebrovascular disease”[Title/Abstract] OR “Cardiovascular Diseases”[Mesh] OR “Heart Diseases”[Mesh] OR “Cerebrovascular Disorders”[Mesh] OR “Stroke”[Mesh]) AND ((“cohort Studies”[Mesh]) OR (cohort [Title/Abstract]) OR (prospective [Title/Abstract]) OR (longitudinal [Title/Abstract]) OR (“Epidemiologic studies”[Mesh]) OR (“case-control studies”[Mesh]) OR OR (Retrospective[Text Word]) OR (Recall*[Text Word]) OR (“Cross-sectional studies”[Mesh]) | 4013 |
| Ischaemic heart disease | ((puberty[Title/Abstract]) OR (menarche[Title/Abstract])) OR (sexual Development[Title/Abstract]) OR (pubertal[Title/Abstract])) OR (spermarche[Title/Abstract]) OR (tanner[Text Word]) OR (voice break*[Text Word]) OR (sexual maturation[Text Word])) AND (“myocardial ischemia”[MeSH Terms] ) OR (“ischaemic heart disease”[ Title/Abstract] OR (“ischaemic heart disease”[Text Word]) AND ((“cohort Studies”[Mesh]) OR (cohort [Title/Abstract]) OR (prospective [Title/Abstract]) OR (longitudinal [Title/Abstract]) OR (“Epidemiologic studies”[Mesh]) OR (“case-control studies”[Mesh]) OR OR (Retrospective[Text Word]) OR (Recall*[Text Word]) OR (“Cross-sectional studies”[Mesh]) | 1250 |
| Breast cancer | ((puberty[Title/Abstract]) OR (menarche[Title/Abstract])) OR (sexual Development[Title/Abstract]) OR (pubertal[Title/Abstract])) OR (spermarche[Title/Abstract]) OR (tanner[Text Word]) OR (voice break*[Text Word]) OR (sexual maturation[Text Word]))AND ((Breast Neoplasm [Mesh]) OR (“Breast cancer”[Title/Abstract]))AND ((“cohort Studies”[Mesh]) OR (cohort [Title/Abstract]) OR (prospective [Title/Abstract]) OR (longitudinal [Title/Abstract]) | 337 |
| Endometrial cancer | ((puberty[Title/Abstract]) OR (menarche[Title/Abstract])) OR (sexual Development[Title/Abstract]) OR (pubertal[Title/Abstract])) OR (spermarche[Title/Abstract]) OR (tanner[Text Word]) OR (voice break*[Text Word]) OR (sexual maturation[Text Word])) AND ((“endometrial neoplasms”[MeSH Terms]) OR (“endometrial cancer”[Title/Abstract])) AND ((“cohort Studies”[Mesh]) OR (cohort [Title/Abstract]) OR (prospective [Title/Abstract]) OR (longitudinal [Title/Abstract]) OR (“Epidemiologic studies”[Mesh]) OR (“case-control studies”[Mesh]) OR OR (Retrospective[Text Word]) OR (Recall*[Text Word]) OR (“Cross-sectional studies”[Mesh]) | 4629 |
| Testicular cancer | ((puberty[Title/Abstract]) OR (menarche[Title/Abstract])) OR (sexual Development[Title/Abstract]) OR (pubertal[Title/Abstract])) OR (spermarche[Title/Abstract]) OR (tanner[Text Word]) OR (voice break*[Text Word]) OR (sexual maturation[Text Word])) AND ((Testicular Neoplasms [MeSH Terms]) OR (testicular cancer [Title/Abstract])) OR (testicular tumor[Title/Abstract])) AND ((“cohort Studies”[Mesh]) OR (cohort [Title/Abstract]) OR (prospective [Title/Abstract]) OR (longitudinal [Title/Abstract]) OR (“Epidemiologic studies”[Mesh]) OR (“case-control studies”[Mesh]) OR OR (Retrospective[Text Word]) OR (Recall*[Text Word]) OR (“Cross-sectional studies”[Mesh]) | 407 |
| Prostate cancer | ((puberty[Title/Abstract]) OR (menarche[Title/Abstract])) OR (sexual Development[Title/Abstract]) OR (pubertal[Title/Abstract])) OR (spermarche[Title/Abstract]) OR (tanner[Text Word]) OR (voice break*[Text Word]) OR (sexual maturation[Text Word])) AND ((“prostatic neoplasms”[MeSH Terms]) OR (“prostate cancer”[ Title/Abstract]) OR (“prostate cancer”[All Fields])) AND ((“cohort Studies”[Mesh]) OR (cohort [Title/Abstract]) OR (prospective [Title/Abstract]) OR (longitudinal [Title/Abstract]) OR (“Epidemiologic studies”[Mesh]) OR (“case-control studies”[Mesh]) OR OR (Retrospective[Text Word]) OR (Recall*[Text Word]) OR (“Cross-sectional studies”[Mesh]) | 156 |
| Depression | ((puberty[Title/Abstract]) OR (menarche[Title/Abstract])) OR (sexual Development[Title/Abstract]) OR (pubertal[Title/Abstract])) OR (spermarche[Title/Abstract]) OR (tanner[Text Word]) OR (voice break*[Text Word]) OR (sexual maturation[Text Word])) AND ((Depression[MeSH Terms] OR (Depression[Title/Abstract]) OR (Depressive Symptoms[Title/Abstract]) OR (Emotional Depression[Title/Abstract]) OR (Depressions”[Title/Abstract]) OR (“Depressive Symptom”[Title/Abstract])) AND ((“cohort Studies”[Mesh]) OR (cohort [Title/Abstract]) OR (prospective [Title/Abstract]) OR (longitudinal [Title/Abstract]) OR (“Epidemiologic studies”[Mesh]) OR (“case-control studies”[Mesh]) OR OR (Retrospective[Text Word]) OR (Recall*[Text Word]) OR (“Cross-sectional studies”[Mesh]) | 1698 |
| Asthma | ((puberty[Title/Abstract]) OR (menarche[Title/Abstract])) OR (sexual Development[Title/Abstract]) OR (pubertal[Title/Abstract])) OR (spermarche[Title/Abstract]) OR (tanner[Text Word]) OR (voice break*[Text Word]) OR (sexual maturation[Text Word]))AND (asthma”[MeSH Terms]) OR (asthma[Title/Abstract]) OR (asthmas[Text Word]) AND ((“cohort Studies”[Mesh]) OR (cohort [Title/Abstract]) OR (prospective [Title/Abstract]) OR (longitudinal [Title/Abstract]) OR (“Epidemiologic studies”[Mesh]) OR (“case-control studies”[Mesh]) OR OR (Retrospective[Text Word]) OR (Recall*[Text Word]) OR (“Cross-sectional studies”[Mesh]) | 362 |

**Table S4. Information on genome-wide association study (GWAS) datasets**

| **Traits name** | **Resource** | **Sample size** | **Reference** | **Download sites** |
| --- | --- | --- | --- | --- |
| Age of menarche | The Reproductive Genetics | 329,245 | Harroud A, Morris JA, Forgetta V, et al. Effect of age at puberty on risk of multiple sclerosis: A mendelian randomization study. | https://www.reprogen.org/Menarche_1KG_NatGen2017_WebsiteUpload.zip |
| Age of voice breaking | CAMBRIDGE UNIVERSITY LIBRARY | 205,354 | Hollis B, Day FR, Busch AS, et al. Genomic analysis of male puberty timing highlights shared genetic basis with hair colour and lifespan. | https://www.repository.cam.ac.uk/bitstreams/c860183c-6d27-411c-9028-8ba7bd2c756e/download |
| Major depression | The Psychiatric Genomics Consortium | case:170,756, control: 329,443 | Howard DM, Adams MJ, Clarke TK, et al. Genome-wide meta-analysis of depression identifies 102 independent variants and highlights the importance of the prefrontal brain regions. | https://datashare.ed.ac.uk/bitstream/handle/10283/3203/PGC_UKB_depression_genome-wide.txt?sequence=3&isAllowed=y |
| Asthma | GWAS Catalog | case: 56,167, control: 352,255 | Valette K, Li Z, Bon-Baret V, et al. Prioritization of candidate causal genes for asthma in susceptibility loci derived from UK Biobank. | https://gwas.mrcieu.ac.uk/datasets/ebi-a-GCST90014325/ |
| Breast cancer | Breast Cancer Association Consortium | case: 133,384, control: 113,789 | Zhang H, Ahearn TU, Lecarpentier J, et al. Genome-wide association study identifies 32 novel breast cancer susceptibility loci from overall and subtype-specific analyses. | https://bcac.ccge.medschl.cam.ac.uk/files/icogs_onco_gwas_meta_overall_breast_cancer_summary_level_statistics.txt.zip |
| Ischemic heart diseases | GWAS Catalog | case: 30,952, control: 187,840 | Not published | https://gwas.mrcieu.ac.uk/datasets/finn-b-I9_ISCHHEART/ |
| Type 2 diabetes | The DIAGRAM consortium | case: 80,154, control: 853,816 | Mahajan A, Spracklen CN, Zhang W, et al. Multi-ancestry genetic study of type 2 diabetes highlights the power of diverse populations for discovery and translation. | http://diagram-consortium.org/downloads.html |
| Endometrial cancer | GWAS Catalog | case: 12,906, control: 108,979 | O'Mara TA, Glubb DM, Amant F, et al. Identification of nine new susceptibility loci for endometrial cancer. | https://gwas.mrcieu.ac.uk/datasets/ebi-a-GCST006464/ |
| Stroke | The Cerebrovascular Disease Knowledge Portal | case: 40,585, control: 406,111 | Malik R, Chauhan G, Traylor M, et al. Multiancestry genome-wide association study of 520,000 subjects identifies 32 loci associated with stroke and stroke subtypes | http://megastroke.org/ |
| Ischemic stroke | The Cerebrovascular Disease Knowledge Portal | case: 34,217, control: 406,111 | Malik R, Chauhan G, Traylor M, et al. Multiancestry genome-wide association study of 520,000 subjects identifies 32 loci associated with stroke and stroke subtypes | http://megastroke.org/ |
| Cardioembolic stroke | The Cerebrovascular Disease Knowledge Portal | case: 7,193, control: 406,111 | Malik R, Chauhan G, Traylor M, et al. Multiancestry genome-wide association study of 520,000 subjects identifies 32 loci associated with stroke and stroke subtypes | http://megastroke.org/ |
| Small-vessel stroke | The Cerebrovascular Disease Knowledge Portal | case: 5,386, control: 406,111 | Malik R, Chauhan G, Traylor M, et al. Multiancestry genome-wide association study of 520,000 subjects identifies 32 loci associated with stroke and stroke subtypes | http://megastroke.org/ |
| Large artery stroke | The Cerebrovascular Disease Knowledge Portal | case: 4,373, control: 406,111 | Malik R, Chauhan G, Traylor M, et al. Multiancestry genome-wide association study of 520,000 subjects identifies 32 loci associated with stroke and stroke subtypes | http://megastroke.org/ |
| Prostate cancer | The PRACTICAL consortium | case: 46,939, control: 27,910 | Schumacher FR, Al Olama AA, Berndt SI, et al. Association analyses of more than 140,000 men identify 63 new prostate cancer susceptibility loci | http://practical.icr.ac.uk/blog/wp-content/uploads/uploadedfiles/oncoarray/MetaSummaryData/meta_v3_onco_euro_overall_ChrAll_1_release.zip |
| Testicular cancer | UK Biobank | case: 797, control: 207,971 | Jiang L, Zheng Z, Fang H, Yang J. A generalized linear mixed model association tool for biobank-scale data. | http://ftp.ebi.ac.uk/pub/databases/gwas/summary_statistics/GCST90041001-GCST90042000/GCST90041906/GCST90041906_buildGRCh37.tsv.gz |
